# DMPC-Based Liposomal Vesicles for Encapsulation and Controlled Release of NMN and Matrigel in Sarcopenia Therapy

**DOI:** 10.3390/ijms26125594

**Published:** 2025-06-11

**Authors:** Alfred Najm, Alexandra Cătălina Bîrcă, Adelina-Gabriela Niculescu, Adina Alberts, Alexandru Mihai Grumezescu, Bianca Gălățeanu, Bogdan Ștefan Vasile, Mircea Beuran, Bogdan Severus Gaspar, Claudiu Ștefan Turculeț, Ariana Hudiță

**Affiliations:** 1 Faculty of Medicine, Carol Davila University of Medicine and Pharmacy, 8 Eroii Sanitari, Sector 5, 050474 Bucharest, Romania; alfred.najm@yahoo.ro (A.N.); adina-magdalena.alberts@rez.umfcd.ro (A.A.); drmirceabeuran@yahoo.com (M.B.); bogdangaspar2005@yahoo.com (B.S.G.); claudiu.turculet@umfcd.ro (C.Ș.T.); 2 Emergency Hospital Floreasca Bucharest, 8 Calea Floreasca, Sector 1, 014461 Bucharest, Romania; 3 Department of Science and Engineering of Oxide Materials and Nanomaterials, National University of Science and Technology Politehnica Bucharest, 011061 Bucharest, Romania; ada_birca@yahoo.com (A.C.B.); adelina.niculescu@upb.ro (A.-G.N.); bogdan.vasile@upb.ro (B.Ș.V.); 4 Research Institute of the University of Bucharest—ICUB, University of Bucharest, 050657 Bucharest, Romania; bianca.galateanu@bio.unibuc.ro (B.G.); ariana.hudita@bio.unibuc.ro (A.H.); 5 Department of Biochemistry and Molecular Biology, Faculty of Biology, University of Bucharest, 050657 Bucharest, Romania

**Keywords:** sarcopenia, liposomes, 1,2-dimyristoyl-sn-glycero-3-phosphocholine (DMPC), nicotinamide mononucleotide (NMN), Matrigel

## Abstract

Accurate diagnosis of diseases in patients is crucial, particularly in older individuals, where the focus is often placed primarily on advanced age and its associated symptoms. However, advancements in technology and research have revealed that certain diseases traditionally linked to aging can also manifest in younger populations, demonstrating similar bodily changes. One such condition is sarcopenia, a degenerative disease of skeletal muscle that arises from various pathological processes affecting the tissues. In this study, we developed a liposomal formulation based on 1,2-dimyristoyl-sn-glycero-3-phosphocholine (DMPC), in which both nicotinamide mononucleotide (NMN) and Matrigel (Mgel) were co-encapsulated, each playing a distinct role in the management of sarcopenia. NMN is known to stimulate the increase of NAD+ levels, while Matrigel enhances the activity of satellite cells, thereby facilitating muscle fiber regeneration and stabilizing protein levels. Results from the DLS, SEM, and TEM analyses revealed significant differences attributed to the type of therapeutic agent used and the synthesis parameters. Additionally, the drug release profile underscored the complementary nature and significance of selecting the appropriate active substances for effective treatment strategies. The in vitro investigations aimed to assess the potential of DMPC lipid vesicles loaded with NMN, either alone or in combination with Matrigel, to counteract sarcopenia-associated oxidative stress and mitochondrial dysfunction. The results showed that both NMN-based formulations reduced oxidative damage, preserved mitochondrial function, and maintained cytoskeletal integrity in a hydrogen peroxide-induced model of sarcopenia. Importantly, the formulation containing both NMN and Matrigel demonstrated superior protective effects, suggesting a synergistic role of the extracellular matrix components in enhancing muscle cell resilience. These findings support the use of DMPC-based delivery systems as promising candidates for sarcopenia therapy and warrant further investigation into their mechanisms of action in preventing muscle cell degeneration.

## 1. Introduction

Numerous health conditions, particularly those associated with aging, directly correlate with the body’s physiological processes. However, these same conditions can also manifest in younger individuals, especially if their bodies exhibit characteristics typically associated with aging. One such condition is sarcopenia, which involves a decline in both muscle mass and muscle strength [1,2,3]. When sarcopenia was first recognized, designated as a disease by the World Health Organization, and assigned the International Classification of Diseases (ICD code M62.8) [4], it was primarily associated with elderly individuals. However, the classification has since expanded to include primary sarcopenia, commonly linked to aging, and secondary sarcopenia, which can occur independently of age and is often associated with other diseases or contributing factors [5,6,7]. Today, it is estimated that over 50 million people are affected by sarcopenia, and projections indicate that this number could exceed 200 million within the next 40 years [5,8].

Sarcopenia encompasses a wide range of potential causes, making it difficult to establish a universal standard for diagnosing the condition in all patients. However, several factors can be analyzed to aid in the diagnosis. Age significantly impacts the muscle tissue affected by sarcopenia, leading to negative effects on homeostasis and disrupting the balance between anabolic and catabolic processes in protein production. As a result, muscle fibers are diminished both quantitatively and qualitatively, and there is a decline in satellite cells, which are crucial for the replacement and regeneration of damaged muscle fibers. Additionally, satellite cells may exhibit dysfunction due to changes in the muscle stem cell niche and factors such as transforming growth factor-beta and myogenin, which are linked to satellite cell regulation. The senescence of satellite cells is particularly associated with sarcopenia, as the accumulation of senescent cells in muscles reduces their regenerative capacity [9,10,11,12]. Moreover, fibroblast growth factors play a vital role in the self-renewal of satellite cells and contribute to the repair of traumatized muscle tissue. Another consequence of sarcopenia is the degradation of proteins, which affect muscle mass through an imbalance between synthesis and degradation processes [13,14,15,16]. Furthermore, genetic factors may also play a role in the development of sarcopenia, influencing both the muscle fiber composition and metabolism associated with growth [17].

**Figure 1:**
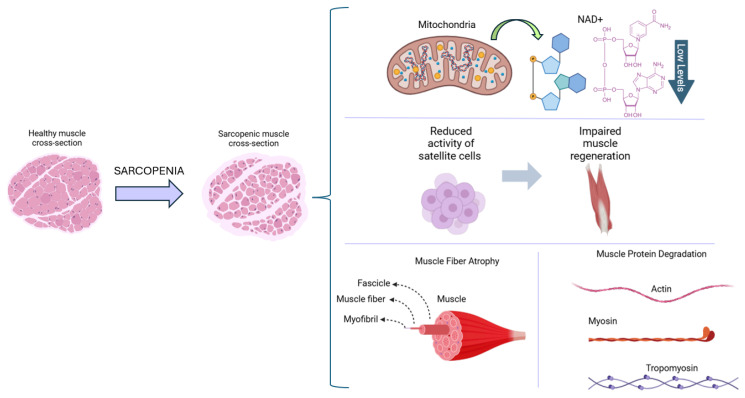
Schematic of the cross-sectional representation of healthy and sarcopenic muscle, illustrating the pathology of sarcopenia. The condition is associated with low NAD⁺ levels, diminished satellite cell activity leading to impaired muscle regeneration, muscle fiber atrophy, and increased muscle protein degradation.

Mitochondria are specialized organelles crucial for energy production, play a key role in free radical metabolism, and are integral to the process of programmed cell death (apoptosis). Mitochondrial dysfunction can arise from various factors; it is not only a consequence of aging, but also a contributing cause of sarcopenia. Studies have shown that muscle mass and its associated functions are closely linked to a healthy number of mitochondria as well as their function and morphology [18,19,20]. In cases of sarcopenia, several mitochondrial dysfunctions have been identified including the uncontrolled accumulation of reactive oxygen species (ROS). Research into the types of ROS involved in the pathophysiology of sarcopenia consistently indicates that hydrogen peroxide (H_2_O_2_) is the predominant species in affected skeletal muscle tissue. Although mitochondrial dysfunction in aging muscle leads to the increased production of superoxide radicals, these are rapidly converted into H_2_O_2_ by superoxide dismutase. Unlike other more reactive ROS, H_2_O_2_ can readily diffuse across cellular and mitochondrial membranes without the need for specialized transport channels. Despite its lower reactivity, its high membrane permeability allows it to disrupt redox homeostasis and contribute to cumulative cellular damage. Therefore, controlling the H_2_O_2_ levels is essential in managing the oxidative stress associated with sarcopenia [15,21,22]. This imbalance between oxidants and antioxidants damages vital molecules such as proteins, DNA, sugars, and lipids, leading to degeneration, atrophy, and muscular dysfunction. Concurrently, oxidative stress negatively impacts mitochondrial biogenesis, resulting in decreased ATP production and subsequent energy metabolism disorders in skeletal muscle fibers [23,24,25,26]. Recent research has highlighted a significant process associated with mitochondrial dysfunction: low levels of NAD+. In healthy skeletal muscle under normal physiological conditions, NAD+ concentrations range from 100 to 1000 µM, maintained by optimal mitochondrial and cytosolic function. In mammals, two key precursors of NAD+ (nicotinamide mononucleotide (NMN) and nicotinamide riboside) are converted into NAD+ through metabolic processes. Consequently, the cascade of mitochondrial dysfunction, characterized by increased oxidative stress, DNA and protein damage, and heightened inflammation, contributes to elevated NAD+ consumption and diminished levels. Notably, NAD+ is one of the most critical metabolites that decline with aging [25,27,28,29,30]. Figure 1 provides a schematic representation of the pathological changes associated with sarcopenia.

**Figure 2:**
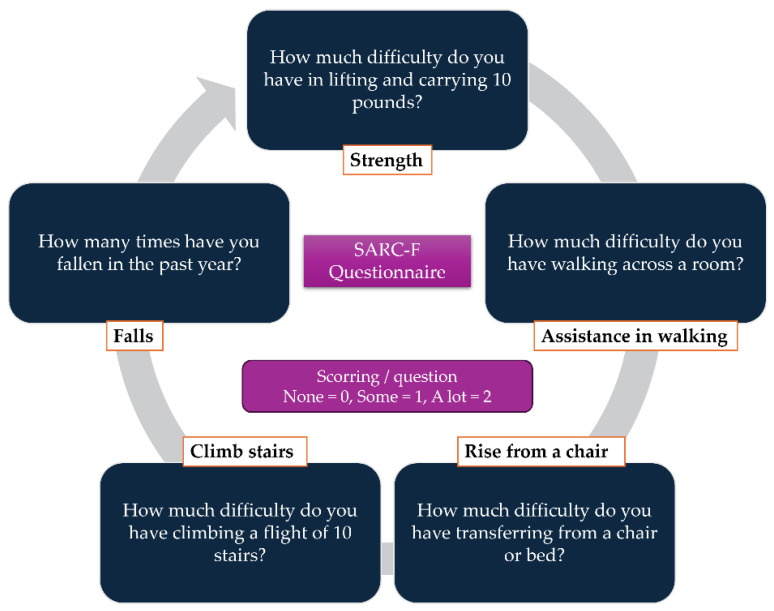
SARC-F questionnaire for the rapid diagnostic of sarcopenia [22].

When it comes to diagnosing sarcopenia, several screening methods and tools are available for patients. In 2012, Malmstrom et al. developed the SARC-F questionnaire, a simple tool designed for the rapid assessment of sarcopenia (Figure 2). This self-reported questionnaire evaluates key aspects of muscle function including muscle strength, the ability to rise from a chair, walking without assistance, climbing stairs, and the incidence of falls. The questionnaire can be seen in Figure 2. In addition to self-reporting tools, various medical imaging techniques can aid in the diagnosis of sarcopenia. For instance, bioelectrical impedance analysis (BIA) provides valuable information about fat-free body mass and total body water. Dual-energy X-ray absorptiometry (DXA) is also used to evaluate bone tissue, allowing for correlations that estimate fat and lean mass; however, its accuracy is somewhat limited. In contrast, computed tomography (CT) and magnetic resonance imaging (MRI) are considered the most reliable imaging modalities for diagnosing sarcopenia. Both techniques generate cross-sectional images, enabling the precise measurements of muscle, fat, and lean mass, with MRI even allowing for detailed assessments of muscle composition. Recently, ultrasound has emerged as a promising tool for evaluating sarcopenia, as it can effectively provide vital information about the muscle characteristics [4,8,31,32].

As of now, there is no specific drug approved for the treatment of sarcopenia, and the primary focus remains on promoting physical activity, with exercise programs tailored to the individual characteristics of patients. Nevertheless, the growing interest in this area has spurred research into pharmacological options for managing sarcopenia. This includes exploring existing medications approved for other conditions as well as investigating new formulations based on recent molecular advancements [12,33,34,35].

This research study investigated a pharmacotherapeutic approach aimed at enhancing the quality of skeletal muscles affected by sarcopenia. It focused on the development and testing of lipid vesicles composed of 1,2-dimyristoyl-sn-glycero-3-phosphocholine (DMPC), which encapsulate nicotinamide mononucleotide (NMN) and Matrigel to create a controlled release system. The choice of a liposomal structure was based on their advantageous properties compared with other delivery systems, notably the similarity of their phospholipid bilayer to cellular membranes. This similarity, along with their biocompatibility, non-immunogenic nature, ability to self-assemble, and capacity to retain and deliver various molecular entities, made them an ideal option. Furthermore, liposomes can be eliminated from the body without affecting renal function [36,37,38,39].

DMPC is a synthetic phospholipid commonly utilized in the formulation of liposomes for medical applications, including drug encapsulation, membrane construction, and drug delivery systems. It is also used in vaccine development, with a melting point (Tm) of 23.6 °C [40,41,42,43]. The use of NMN in this study was grounded in research showing that one of the most significant “symptoms” of sarcopenia is a reduction in NAD+ levels. By restoring the NAD⁺ levels using NMN, a central cofactor involved in numerous metabolic pathways that support mitochondrial respiration, DNA repair, and cellular homeostasis, this enhances mitochondrial function, which in turn supports muscle regeneration and facilitates metabolic reprogramming. As NMN serves as a precursor to NAD+ and has demonstrated promising results in previous studies aimed at addressing sarcopenia, it was utilized in the design of the liposomal delivery system presented in this research [36,44,45,46]. Additionally, sarcopenia adversely affects satellite cells and reduces the levels of crucial growth factors necessary for muscle function and strength. Therefore, another key component of the developed delivery system is Matrigel, which consists of a mixture of proteins, proteoglycans, and various growth factors. This solubilized basement membrane protein is extensively used in medical applications and is incorporated into the material development for drug delivery systems from both engineering and biological perspectives [47,48,49,50].

**Figure 3:**
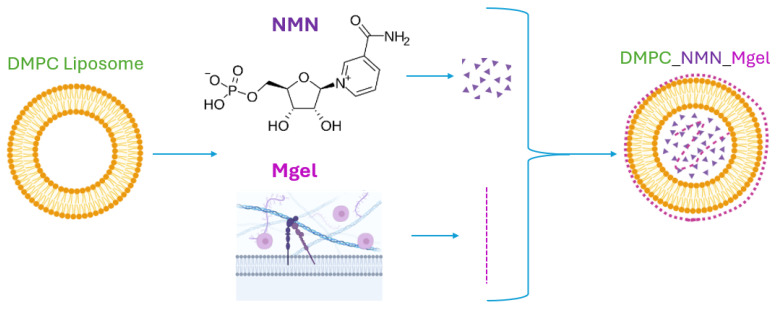
Drug delivery system design of DMPC liposomes that encapsulate NMN and Matrigel for sarcopenia treatment approach.

Therefore, the liposomal formulation developed in this study represents a therapeutic strategy targeting sarcopenia-affected cells. NMN acts on intracellular metabolic deficiencies by restoring the NAD⁺ levels, while Matrigel supports satellite cells and matrix environment remodeling. While each component individually contributes therapeutic effects by targeting specific alterations induced by sarcopenia, the multifactorial nature of this condition requires a multifaceted approach. The combined action of multiple agents synergistically enhances functional muscle recovery by concurrently addressing diverse pathological processes, thereby providing a more effective and comprehensive therapeutic strategy. A schematic representation of the NMN and Matrigel co-delivery system using the DMPC_NMN_Mgel liposomal formulation is shown in Figure 3.

The study assessed the liposomes from a physicochemical standpoint, examining the correlation between their characteristics and biological effects. In vitro experiments involved C2C12 cells differentiated into myotubes, which were treated to induce muscle atrophy. The efficacy of liposomes encapsulating NMN and Matrigel were evaluated under conditions of sarcopenia, with the aim of establishing them as a potential future solution for the reduction and treatment of this condition.

## 2. Results

Following the preparation of DMPC-based liposomes encapsulating both NMN, Matrigel, and their mix, the samples were analyzed from a physicochemical point of view in order to establish the characteristics of the liposomes in terms of stability in liquid, hydrodynamic and physical size, and morphostructure as well as the observation of the existence of an intact membrane or its defects. These characteristics are critical as they correlate with the biological responses of controlled release systems and provide physicochemical confirmation of the success of the synthesis process. Initially, the samples were analyzed using dynamic light scattering with PBS as the analysis solvent, simulating the physiological conditions as closely as possible. The results are presented both graphically and in tabular form for a comprehensive overview of the data obtained.

**Figure 4:**
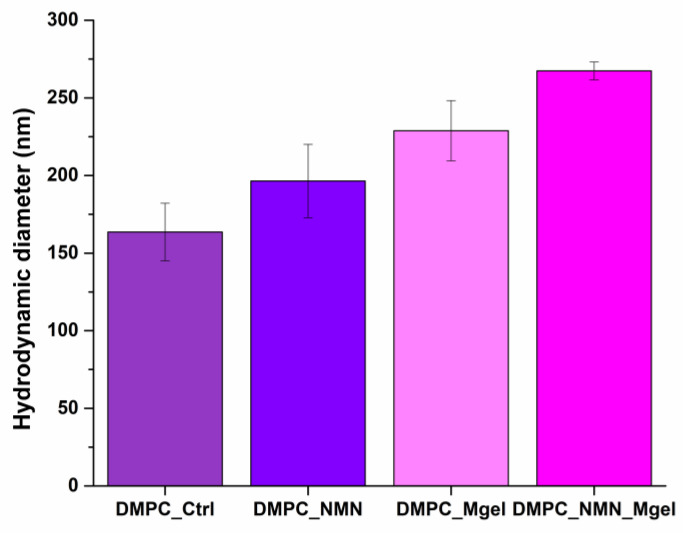
DLS result expressed as hydrodynamic diameter (nm) for DMPC_Ctrl, DMPC_NMN, DMPC_Mgel, DMPC_NMN_Mgel.

Figure 4 and Table 1 present the experimental data from the DLS analysis, detailing the hydrodynamic diameters for the samples DMPC_Ctrl, DMPC_NMN, DMPC_Mgel, and DMPC_NMN_Mgel. The recorded hydrodynamic diameters ranged from 163.6 nm to 267.4 nm, showing a gradual increase from the control sample (DMPC_Ctrl) to the sample that encapsulated both therapeutic substances (DMPC_NMN_Mgel). This trend suggests that the presence of the different encapsulated substances affects the liposome core and their overall chemical properties, resulting in varying hydrodynamic diameters among the samples. The observed increase from DMPC_Ctrl to DMPC_NMN_Mgel corroborates the successful encapsulation of the therapeutic mix, with DMPC_NMN_Mgel exhibiting the largest hydrodynamic diameter among the liposomes tested.

**Figure 5:**
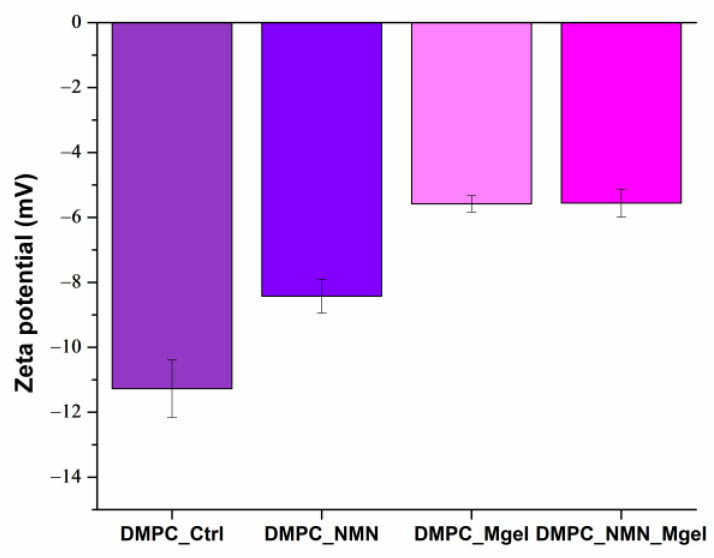
DLS result expressed as zeta potential (mV) for DMPC_Ctrl, DMPC_NMN, DMPC_Mgel, and DMPC_NMN_Mgel.

The DLS results for the zeta potential of the analyzed samples are presented both graphically in Figure 5 and numerically in Table 2. The data revealed that the DMPC_Ctrl sample exhibited the highest zeta potential, recorded at −11.27 mV, while the DMPC_NMN_Mgel sample showed the lowest value at −5.55 mV. Interestingly, this trend was the opposite to that observed for the hydrodynamic diameter. All samples displayed a negative electrical charge on the liposome surface, and the obtained values indicated moderate stability, which diminished with the incorporation of therapeutic substances. This finding is consistent with the hydrodynamic diameter results: the control sample DMPC_Ctrl, which had the smallest hydrodynamic diameter, also possessed the highest zeta potential. This suggests that the addition of therapeutic agents may introduce chemical changes that contribute to the instability of the suspensions, potentially leading to liposome aggregation. Additionally, the physical size variations could further influence the zeta potential.

Conversely, Table 3 provides the values for the polydispersity index, which categorized all four samples analyzed as monodisperse. This indicated a consistent particle size distribution, reflecting the stability of the liposomes in PBS. These findings align with the desired characteristics for the controlled release system we are developing and investigating.

**Figure 6:**
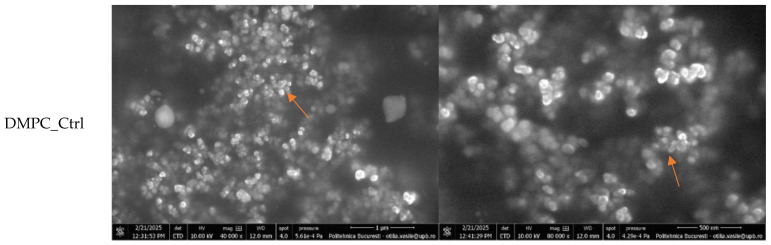

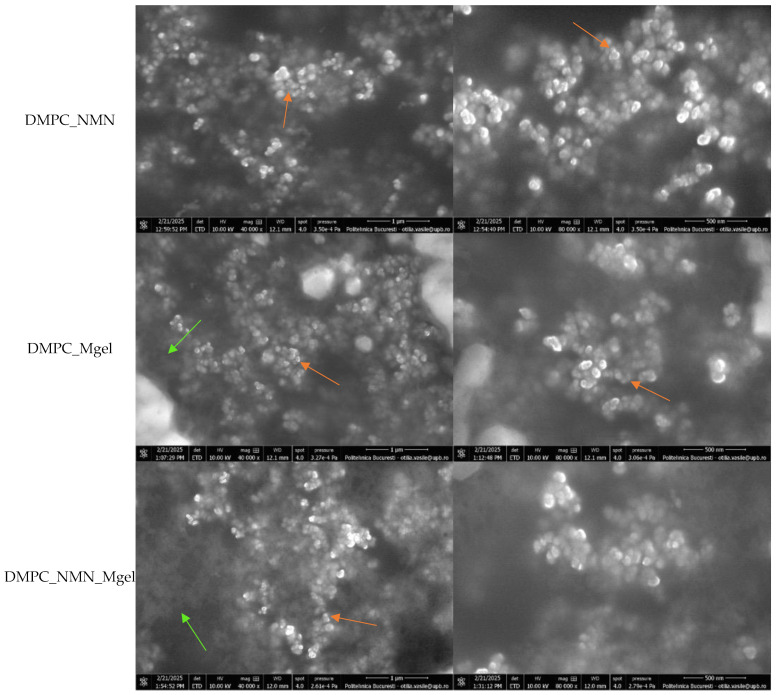
SEM micrographs obtained for the liposomal formulations: DMPC_Ctrl (liposomes without active compounds), DMPC_NMN (DMPC liposomes with nicotinamide mononucleotide), DMPC_Mgel (DMPC liposomes with Matrigel), and DMPC_NMN_Mgel (combined formulation). Orange arrows indicate liposomes, while green arrows indicate Matrigel structures. All formulations were based on DMPC vesicles.

Subsequently, the samples were examined using electron microscopy to gather important information regarding the physical size and morphology of the liposomes. Figure 6 displays the SEM micrographs of the DMPC_Ctrl, DMPC_NMN, DMPC_Mgel, and DMPC_NMN_Mgel samples, captured at magnifications of 40,000× and 80,000×.

All micrographs confirmed the successful formation of numerous lipid vesicles across all four samples. The vesicles predominantly exhibited a spherical morphology and small sizes, contributing to their natural tendency to aggregate, a phenomenon observed in all cases. The encapsulation of NMN (DMPC_NMN) did not induce noticeable structural changes compared with the control sample (DMPC_Ctrl). However, the addition of Matrigel introduced certain modifications, as evidenced by the micrographs of the DMPC_Mgel and DMPC_NMN_Mgel samples. First, the sample observation process became more challenging due to the complex structure of Matrigel. Additionally, distinct Matrigel networks were visible in regions lacking lipid vesicles, indicating the presence of non-encapsulated material. Furthermore, a reduction in liposome size was observed, suggesting that Matrigel may influence the vesicle formation and size distribution. Despite these variations, all four liposome formulations consistently demonstrated the successful formation of numerous small lipid vesicles.

**Figure 7:**
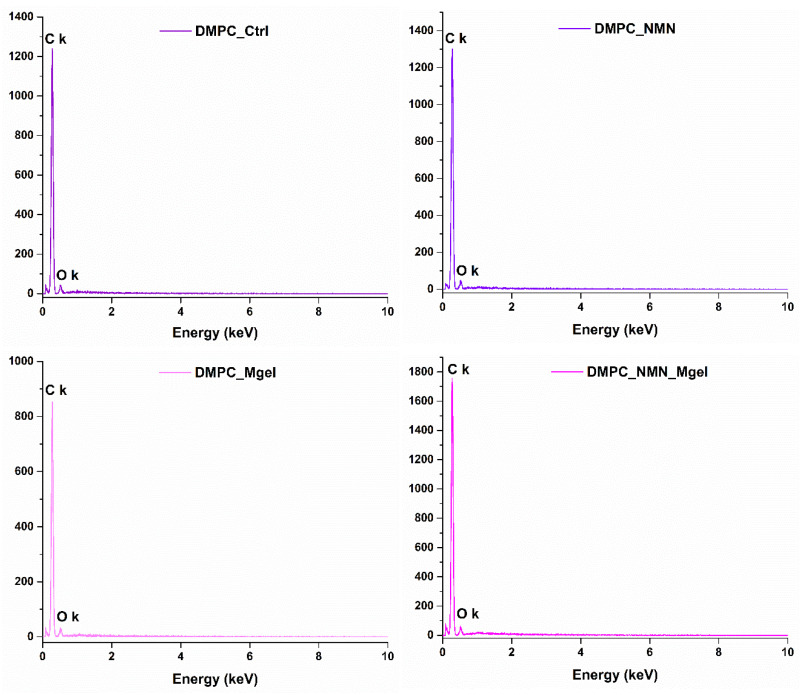
EDS spectra obtained for DMPC_Ctrl, DMPC_NMN, DMPC_Mgel, and DMPC_NMN_Mgel.

The EDS module of the electron microscope was utilized to obtain the characteristic EDS spectra (Figure 7) for each of the four liposome formulations. Given the organic nature of the substances involved in the formulation of DMPC-based liposomes containing NMN and Matrigel, the identified elements were carbon and oxygen, with no impurities detected in the samples.

**Figure 8:**
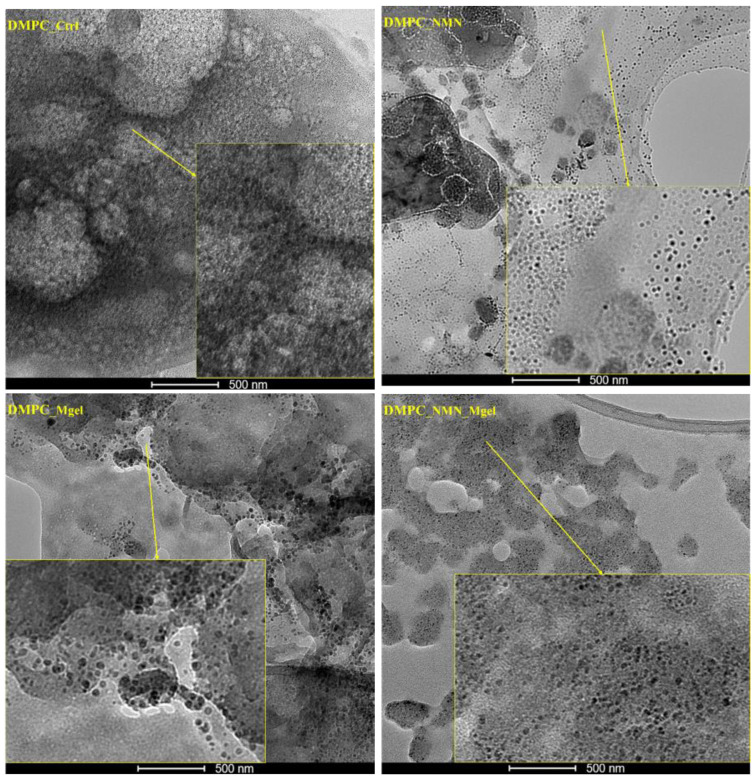
TEM micrographs obtained for DMPC_Ctrl, DMPC_NMN, DMPC_Mgel, and DMPC_NMN_Mgel.

The small dimensions of the liposomes observed in the SEM micrographs, coupled with the challenges of assessing their morphologies, prompted further investigation of the DMPC_Ctrl, DMPC_NMN, DMPC_Mgel, and DMPC_NMN_Mgel samples using a transmission electron microscope for higher resolution imaging (Figure 8). The control sample, DMPC_Ctrl, displayed a multitude of lipid vesicles that were evenly distributed and exhibited intact membranes, confirming the successful formation of the control liposomes. In the case of the NMN-encapsulated liposomes (DMPC_NMN), the individual vesicles were clearer and more distinct, allowing for a detailed observation of their well-defined spherical membranes. The morphological characteristics of the DMPC_Mgel liposomes remained consistent with previous observations; however, they displayed noticeable morphological and dimensional non-uniformity among the numerous lipid vesicles identified. For the DMPC_NMN_Mgel therapeutic mix, the presence of numerous individual vesicles with intact membranes was also observed. Their morphology fell between the well-defined structure seen in DMPC_NMN and the non-uniformity observed in DMPC_Mgel, suggesting that NMN may act as a stabilizer in addition to its therapeutic role, contributing to the formation of liposomes with more clearly defined morphologies.

**Figure 9:**
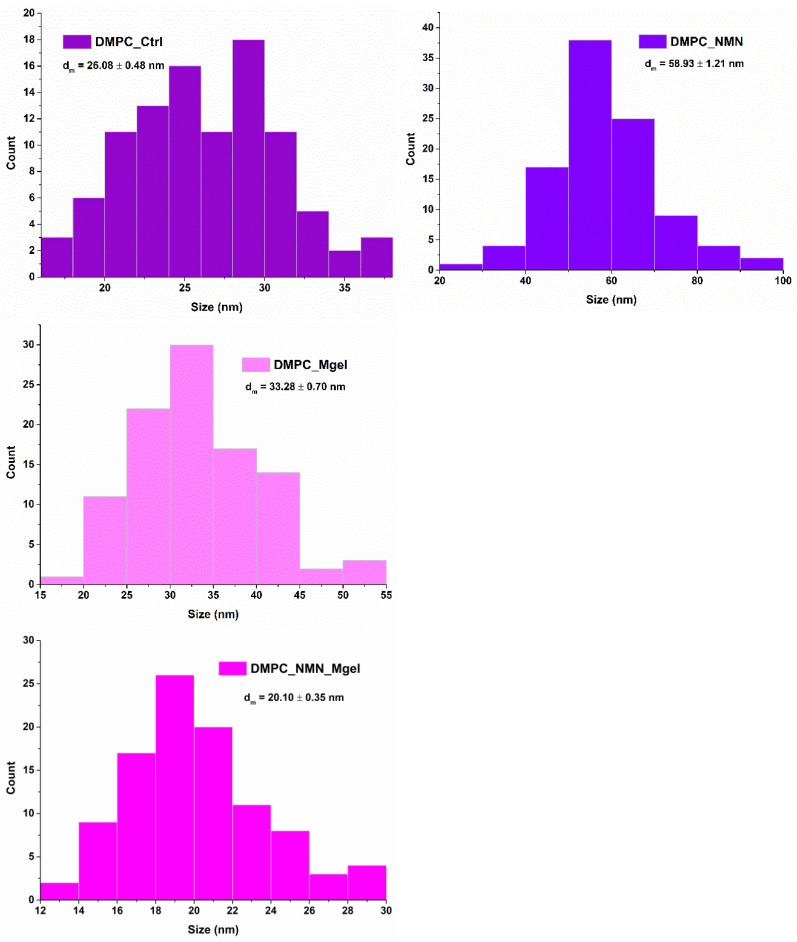
Vesicle size distribution of DMPC_Ctrl, DMPC_NMN, DMPC_Mgel, and DMPC_NMN_Mgel. Data are presented as the mean ± standard deviation.

High-resolution imaging of the liposomes enabled precise size measurements and the generation of size distribution histograms. Figure 9 illustrates the monomodal distribution obtained for all samples, confirming the nanometric dimensions of DMPC_Ctrl, DMPC_NMN, DMPC_Mgel, and DMPC_NMN_Mgel. Among the samples, the DMPC_NMN liposomes exhibited the largest average vesicle size (58.93 nm), whereas the DMPC_NMN_Mgel sample presented the smallest average size (20.10 nm). Notably, the presence of NMN appeared to influence the vesicle dimensions, as both NMN-containing formulations were positioned at opposite extremes of the size spectrum. Additionally, the therapeutic mix (DMPC_NMN_Mgel) exhibited a synergistic effect, contributing to a well-defined liposomal morphology and a reduction in overall vesicle size.

**Figure 10:**
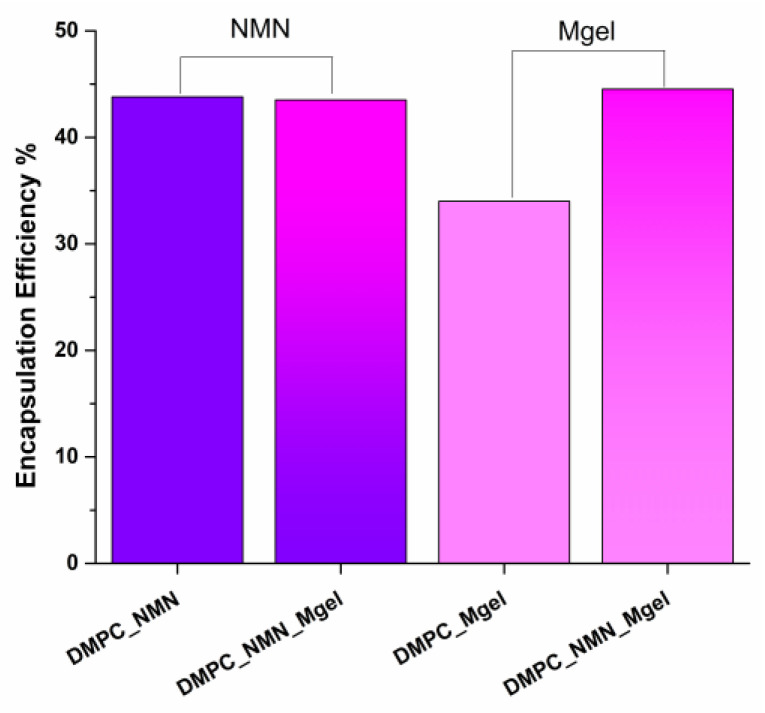
Encapsulation efficiency of NMN and Matrigel in DMPC-based lipid vesicles (the first two columns indicate the NMN encapsulation, and the third and the fourth column indicate the Mgel encapsulation).

**Figure 11:**
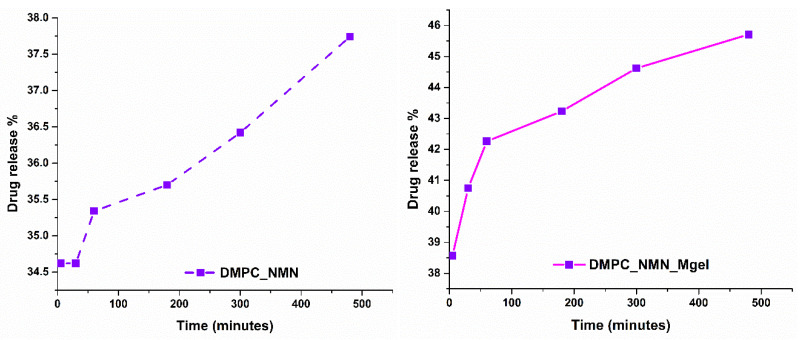

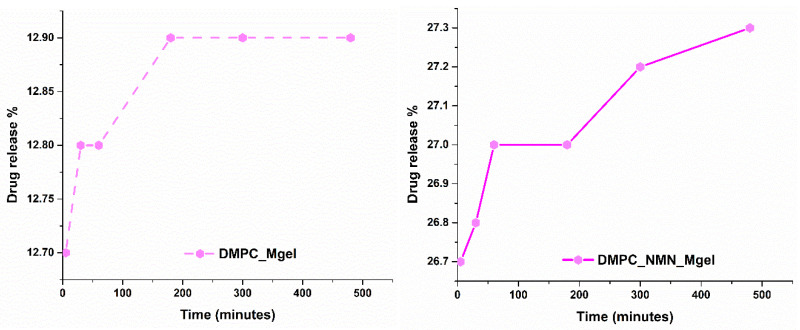
Time-dependent drug release of NMN and Mgel from DMPC_NMN, DMPC_Mgel, and DMPC_NMN_Mgel. (**Left**) Result for NMN and Mgel from the one therapeutic compound samples (DMPC_NMN and DMPC_Mgel), and (**right**) result for NMN and Mgel from the therapeutic mix sample (DMPC_NMN_Mgel).

In the context of this research study, we evaluated the DMPC_NMN, DMPC_Mgel, and DMPC_NMN_Mgel liposomes to gather insights on the encapsulation efficiency (Figure 10) and time-dependent drug release rates (Figure 11). For the DMPC_NMN and DMPC_Mgel samples, each therapeutic substance was assessed individually. In contrast, the DMPC_NMN_Mgel sample, which contained both NMN and Matrigel, allowed for the separate evaluation of each component, resulting in two sets of data for the encapsulation efficiency and drug release rates over time for this sample.

The encapsulation efficiency of substances is influenced by their specific characteristics. In this study, we focused on NMN, a naturally occurring nucleotide that serves as a precursor to NAD+, which comprises three structural components: nicotinamide, ribose sugar, and a phosphate group. We also examined Matrigel, a biological compound derived from the extracellular matrix of Engelbreth-Holm-Swarm mouse sarcoma cells, which consists of proteins (such as laminin, collagen, and entactin), proteoglycans (like heparan sulfate proteoglycans), and various growth factors (including EGF, TGF-β, bFGF, and IGF-1). In terms of NMN encapsulation, samples DMPC_NMN and DMPC_NMN_Mgel achieved an encapsulation rate of approximately 40%. This indicated a positive correlation between the efficient encapsulation process and the presence of DMPC in combination with NMN. Conversely, Matrigel alone, as assessed in the sample DMPC_Mgel, demonstrated a lower encapsulation rate of about 30%. However, with the addition of NMN in the DMPC_NMN_Mgel sample, the encapsulation rate was similar to that of NMN alone, around 40%. These results suggest that NMN may play a facilitating role in improving the encapsulation efficiency of Matrigel, enabling a more effective incorporation of the biological matrix into the lipid vesicles compared with when Matrigel was encapsulated alone.

The evaluation of the drug release rate across all samples clearly demonstrated a time-dependent release profile, indicating that these systems provide a controlled release mechanism for NMN and Matrigel. In the DMPC_NMN formulation, an initial burst release occurred within the first few minutes, reaching approximately 34%. The release continued to increase gradually over time, but with minimal variation, reaching approximately 38% after eight hours. In the DMPC_NMN_Mgel formulation, a similar release pattern was observed, but with higher release values. The initial burst release reached 39%, progressively increasing to 46% after eight hours.

Compared with NMN, the Matrigel-loaded formulations exhibited lower release rates and smaller variations over time, suggesting a more sustained release. In the DMPC_Mgel formulation, Matrigel was released at a rate of 12.7% within the first minutes, increasing only slightly to 12.9% at the end of the eight hour test period. In contrast, the DMPC_NMN_Mgel formulation showed a more pronounced initial burst release of 26.7%, which gradually increased to 27.3% after eight hours.

By correlating these findings with encapsulation efficiency, the results indicate a complementary effect between NMN and Matrigel in terms of release behavior. The incorporation of both NMN and Matrigel within the same formulation (DMPC_NMN_Mgel) led to an improved Matrigel release profile, facilitated by the presence of NMN. These results suggest that NMN may enhance the diffusion and release kinetics of Matrigel, contributing to a more efficient and sustained release system.

**Figure 12:**
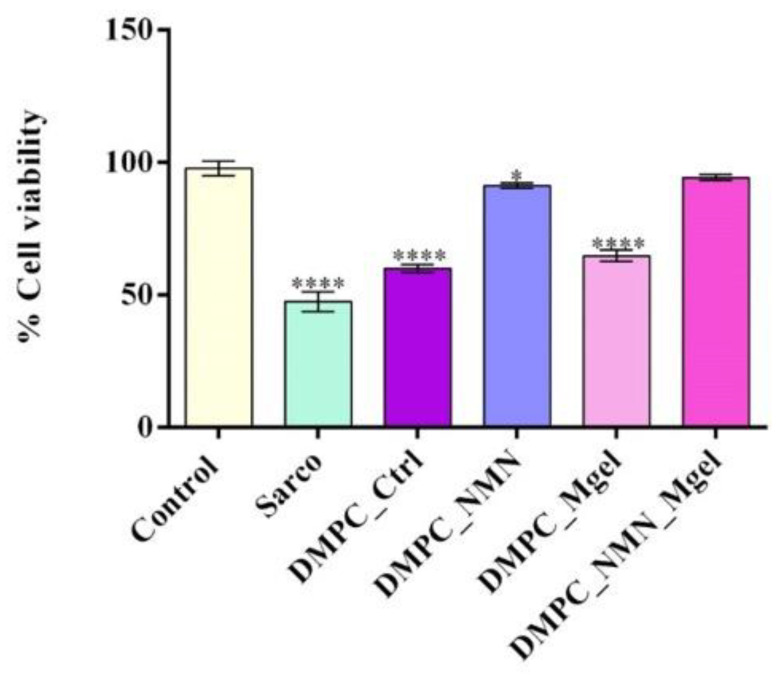
Cell viability of the C2C12 myotubes assessed by the MTT assay after 48 h of treatment under the tested conditions. Besides the experimental control, for all conditions, H_2_O_2_ was added to induce muscular atrophy in the C2C12 myotube cultures. Data are presented as the mean ± SD. *p*-values are indicated as follows: *p* ≤ 0.05 (*) and *p* ≤ 0.0001 (****) compared with the control group. To assess the oxidative stress under the tested conditions, the intracellular reactive oxygen species (ROS) levels were quantified (Figure 13). As expected, the C2C12 myotubes exposed to H_2_O_2_ presented a statistically significant elevation in ROS production in comparison with the experimental control, reflecting the chronic oxidative stress associated with sarcopenia. The DMPC_Ctrl group also exhibited high ROS levels compared with the experimental control, indicating that the bare lipid vesicles may not provide antioxidative benefits. Likewise, the addition of Matrigel (DMPC_Mgel) to the vesicle formulation did not reduce ROS production under sarcopenic conditions, as the values remained similar to those observed in the DMPC_Ctrl group. In contrast, both DMPC_NMN and DMPC_NMN_Mgel were highlighted as effective tools in reducing ROS production, with both groups presenting a statistically significant decrease in ROS production compared with the H_2_O_2_-treated group. However, no significant differences were observed between the two NMN-loaded formulations, suggesting that the NMN was primarily responsible for the reduction in oxidative stress.

To mimic sarcopenia in vitro, C2C12 cells differentiated into myotubes were treated with H_2_O_2_ alone or in combination with the tested lipid vesicles. The colorimetric MTT assay was employed to quantitatively assess the cell viability post-treatment (Figure 12). After 48 h, exposure to sarcopenia-mimicking conditions (Sarco group) resulted in a statistically significant reduction in cellular metabolic activity relative to the untreated control. Treatment with pristine lipid particles (DMPC_control) failed to mitigate the negative impact of H_2_O_2_ on cell viability, as the DMPC-treated myotubes showed a statistically significant decrease in cell viability in comparison with the untreated control. These results suggest that the lipid vesicles alone lacked bioactivity in the context of sarcopenic conditions. In contrast, the formulation loaded with NMN (DMPC_NMN) triggered a partial restoration of the cell viability to levels approaching those of the control, suggesting that the active compound ameliorated sarcopenia-induced damage. Moreover, the lipid vesicles loaded with Matrigel (DMPC_Mgel) failed to improve the C2C12 myotube cell viability, which remained statistically significantly reduced compared with the experimental control. The most pronounced improvement in cell viability was observed in the DMPC_NMN_Mgel group, where the cell viability of the C2C12 myotubes exposed to sarcopenia-mimicking conditions was restored to levels similar to the control C2C12 myotubes. This highlights the novel formulation’s capacity to restore cellular metabolic activity, suggesting a potential synergic effect between NMN and Matrigel.

**Figure 13:**
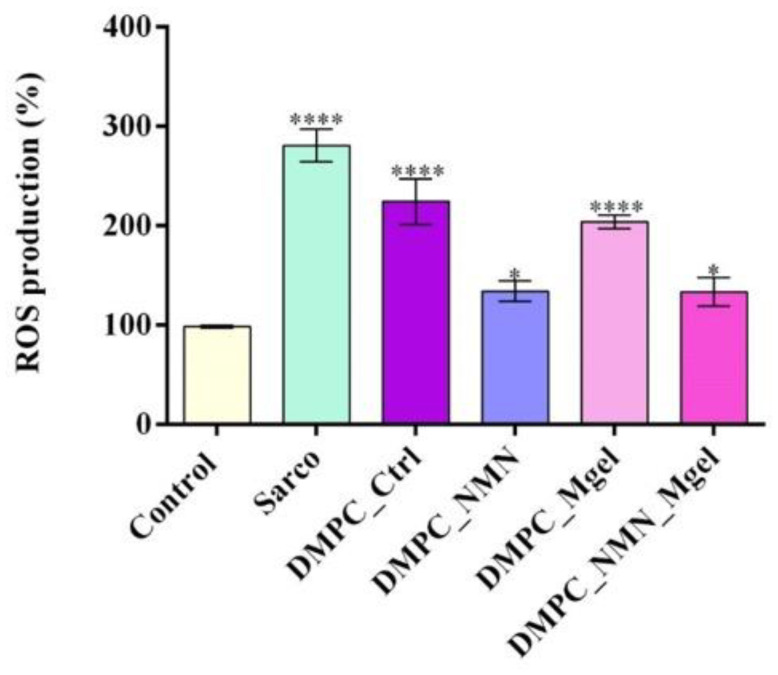
ROS production in the C2C12 myotube cultures after 48 h of treatment under the tested conditions. Besides the experimental control, for all conditions, H_2_O_2_ was added to induce muscular atrophy in C2C12 myotubes culture. Data are presented as mean ± SD. *p*-values are indicated as follows: *p*-values are indicated as follows: *p* ≤ 0.05 (*) and *p* ≤ 0.0001 (****) compared with the control group.

**Figure 14:**
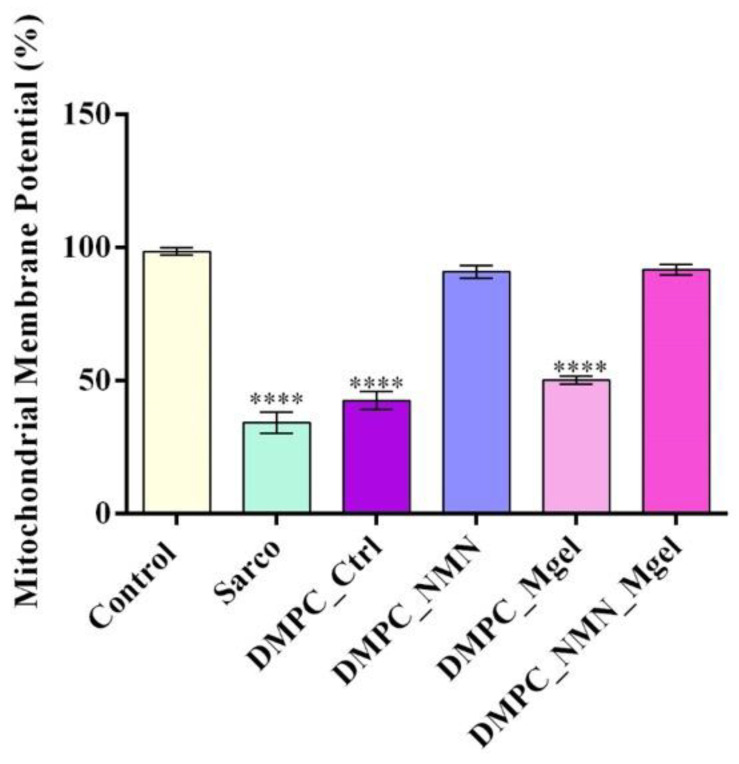
Mitochondrial membrane potential of C2C12 myotubes after 48 h of treatment under the tested conditions. Besides the experimental control, for all conditions, H_2_O_2_ was added to induce muscular atrophy in C2C12 myotubes culture. Data are presented as mean ± SD. *p*-values are indicated as follows: *p* ≤ 0.0001 (****) compared with the control group.

Given the central role of the mitochondria in sarcopenia, the mitochondrial membrane potential (MMP) was assessed as an indicator of mitochondrial integrity (Figure 14). As expected, myotubes subjected to H_2_O_2_ treatment displayed a significant loss of MMP, confirming severe mitochondrial dysfunction and depolarization in response to sarcopenic stress. Treatment with DMPC vesicles alone failed to preserve mitochondrial polarization, with cells still showing statistically significant mitochondrial depolarization compared with the experimental control. In contrast, DMPC_NMN treatment partially restored the MMP, bringing values closer to those of the experimental control group and thus suggesting the drug’s positive impact in preserving mitochondrial function and its overall stabilization. Similar results were also obtained for DMPC_NMN_Mgel formulation, while the DMPC_Mgel did not show a measurable benefit in restoring MMP.

**Figure 15:**
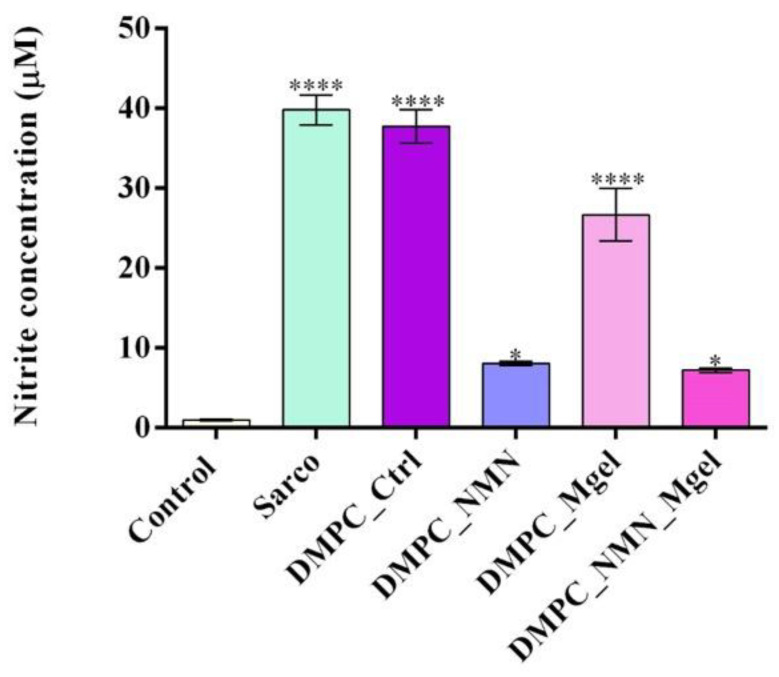
NO production in C2C12 myotubes cultures after 48 h of treatment under the tested conditions. Besides the experimental control, for all conditions, H_2_O_2_ was added to induce muscular atrophy in C2C12 myotubes culture. Data are presented as mean ± SD. *p*-values are indicated as follows: *p*-values are indicated as follows: *p* ≤ 0.05 (*) and *p* ≤ 0.0001 (****) compared with the control group.

Nitric oxide (NO) production reflects muscle atrophy, mitochondrial dysfunction, and inflammation in sarcopenia. For this purpose, the NO production was assessed (Figure 15) and revealed that the NO levels were markedly elevated in sarcopenic and DMPC_Ctrl-treated cells as compared with the experimental control, reflecting inflammatory stress and NO overproduction. Notably, DMPC_NMN and DMPC_NMN_Mgel treatments significantly suppressed NO levels, suggesting anti-inflammatory and cytoprotective effects. As the lipid vector itself exhibited minimal impact on NO production as compared with the experimental control, the obtained results highlight the bioactive roles of loaded NMN and Matrigel components on alleviating the oxidative stress-induced damage by sarcopenia-mimicking conditions.

**Figure 16:**
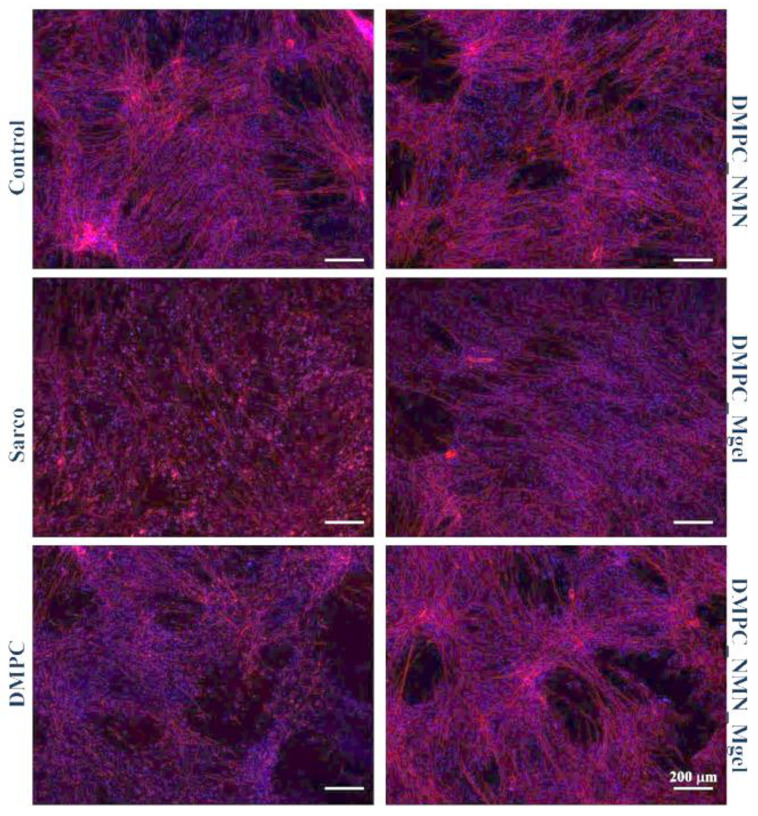
Fluorescence micrographs revealing the F-actin filaments (red) and cell nuclei (blue) of the C2C12 myotubes after 48 h of treatment under the tested conditions. Besides the experimental control, for all conditions, H_2_O_2_ was added to induce muscular atrophy in the C2C12 myotube cultures.

Cytoskeletal integrity was evaluated via fluorescence microscopy using TRITC-phalloidin and DAPI staining to visualize F-actin filaments and nuclei, respectively (Figure 16). Control myotubes displayed elongated, well-aligned F-actin filaments, consistent with the typical cytoskeletal organization of these cells. In contrast, cells exposed to sarcopenia-mimicking conditions exhibited marked cytoskeletal disorganization, characterized by shortened and fragmented F-actin filaments, reduced cell elongation, and overall altered cell morphology, features consistent with sarcopenic-like degeneration. The DMPC_Ctrl group showed similar alterations, suggesting that the pristine lipid vesicles alone failed to preserve the cytoskeletal architecture under sarcopenia-mimicking conditions. Interestingly, the DMPC_Mgel group exhibited a partial restoration of the F-actin filament structure, with some cells displaying more continuous and oriented F-actin filaments compared with the Sarco group. These results suggest that the Matrigel may support structural remodeling and cell adhesion. The DMPC_NMN group showed an evident recovery of the cytoskeletal integrity, with elongated and well-defined F-actin filaments and a more organized filamentous network. Notably, the DMPC_NMN_Mgel group exhibited the most pronounced protective effects among all treatments, with both the cell morphology and F-actin architecture closely resembling those of the experimental control group. These findings reinforce the hypothesis of a synergistic effect between NMN and Matrigel in preserving cytoskeletal integrity under sarcopenia-mimicking conditions.

## 3. Discussion

This study aimed to develop and evaluate DMPC-based liposome pharmaceutical formulations that encapsulated both NMN and Matrigel, targeting a therapeutic approach to sarcopenia, a degenerative disease affecting skeletal muscle. Research has shown that sarcopenia is associated with reduced NAD+ levels and the impaired functionality of satellite cells, which contribute to protein degradation and hinder muscle regeneration. Consequently, this study addressed these critical limitations by utilizing NMN, a precursor of NAD+, alongside Matrigel, a composition of proteins and growth factors. Together, these components hold promise for enhancing muscle health and functionality in individuals affected by sarcopenia.

Physicochemical investigations have allowed for a comprehensive characterization of the therapeutic liposomes, assessing their surface charge, colloidal stability, hydrodynamic and physical diameter, morphology, and drug behavior in terms of encapsulation efficiency and release kinetics. The use of DMPC as the primary lipid component resulted in a negative zeta potential for all of the analyzed samples, a feature that is particularly advantageous for sarcopenia therapy. In biological environments, most cell membranes naturally exhibit a negative charge. Consequently, negatively charged liposomes are less susceptible to rapid clearance by the reticuloendothelial system compared with positively charged counterparts, thereby prolonging their circulation time. Furthermore, positively charged liposomes tend to attract serum proteins, leading to recognition by macrophages, and premature elimination. In contrast, the slightly negative charge enhanced liposomal bioavailability and improved the pharmacokinetics, making them a more effective drug delivery system [51,52,53]. A study by Epstein-Barash et al. [54] demonstrated that liposomes composed of 1,2-distearoyl-sn-glycero-3-phosphocholine (DSPC), anionic distearoyl-phosphatidylglycerol (DSPG), and cholesterol at a molar ratio of 3:1:2, with an average size of 85 nm and a negative charge, exhibited favorable internalization by mononuclear phagocytic system cells. In contrast, positively charged liposomes with larger dimensions triggered cytokine activation and increased toxicity. A study conducted by Alexis Demonbreun et al. [55] indicated that the repair process of muscle membranes was closely associated with interactions involving negatively charged phospholipids including phosphatidylserine. Proteins critical to muscle membrane repair, such as dysferlin, MG53, and annexins, bind to these negatively charged phospholipids, thereby facilitating the healing process and preserving muscle integrity. These findings underscore the preference for slightly negatively charged liposomes in drug delivery systems as they are more readily recognized as biocompatible by macrophages, reducing the risk of an inflammatory response and premature clearance.

An essential characteristic of liposomes in the design of drug delivery systems is their size, as it directly influences therapeutic performance. There is a well-established correlation between liposome size and drug release efficiency. Studies have demonstrated that liposomes within the 50–100 nm range, or even smaller, are particularly suitable for targeted applications. Their reduced size enables them to evade immune system clearance via phagocytosis, thereby extending their circulation time in the bloodstream [56]. In this study, the hydrodynamic diameter of the tested liposomes ranged from 163.6 nm (DMPC_Ctrl) to 267.4 nm (DMPC_NMN_Mgel), with variations in size influenced by the encapsulated components. The average physical size of the liposomes, as determined through microscopy, was smallest for DMPC_NMN_Mgel (20.10 nm), whereas the largest vesicles were recorded in the DMPC_NMN sample. However, the presence of NMN and Matrigel significantly influenced the liposome dimensions. Matrigel showed a lower encapsulation efficiency, likely due to its protein-rich composition, which may lead to partial adsorption onto the liposome surface rather than full incorporation into the aqueous core. NMN, being highly hydrophilic, exhibited a much higher encapsulation efficiency, forming larger vesicles due to its compatibility with DMPC and ability to interact with the lipid bilayer. While TEM provides valuable insight into vesicle morphology, in our study, the functionality and structural integrity of the DMPC_NMN_Mgel system were further supported by the encapsulation efficiency and sustained release behavior of NMN and Matrigel. These results complement the morphological observations and reinforce the distinct membrane vesicle formation of the hybrid liposomal system. Beyond the encapsulated core, the synthesis process itself plays a critical role in determining the vesicle size. In this study, small unilamellar vesicles were obtained, primarily due to the combined effects of temperature and sonication applied during the lipid film hydration process. When lipid hydration occurs above the Tm of DMPC, the lipid enters the liquid crystalline phase, where molecular mobility is significantly increased. This enhanced fluidity allows for more efficient vesicle formation compared with the gel phase, where lipid movement is more restricted [57]. Ultrasound energy effectively reduces the size of the liposomes, as it breaks down larger vesicles into smaller ones through cavitation effects. A study by Camila Fabiano de Freitas et al. [58] demonstrated that after 90 s of sonication of DOPC/F127 liposomes, their size was reduced from 322.9 nm to 61.0 nm, while the temperature increased slightly from 25 °C to 27 °C. The sonication process not only influences the size but also affects the polydispersity index (PDI), which reflects the colloidal stability and size uniformity. In this study, the PDI values remained below 0.1, indicating notable stability and homogeneity of the liposomal dispersion. This finding aligns with research by Fatemeh Nowroozi et al. [59], who demonstrated that the sonication bath used during the hydrating process significantly reduced the PDI, leading to increased uniformity in niosomes. To further refine the liposome size, three post-hydration processing techniques were compared in the study by Nowroozi et al.: extrusion, probe sonication, and high-pressure homogenization. Among these, probe sonication yielded the smallest vesicle size, as confirmed by DLS analysis, making it the most effective technique for downsizing lipid vesicles to nanoscale dimensions.

Over time, liposomes have emerged as one of the most effective and viable solutions in controlled drug release, particularly in two key areas: drug delivery and gene therapy. Among the various routes of administration, intravenous and intramuscular formulations remain the most widely used, while oral liposomal delivery is currently being explored as a promising alternative. Regulatory advancements in liposomal drug formulations have also been made, with the U.S. Food and Drug Administration (FDA) introducing a preliminary draft in 2002 titled “Guidance for Industry on Liposome Drug Products”, which was later expanded into a more comprehensive document in 2015, entitled “Liposome Drug Products Chemistry, Manufacturing, and Controls; Human Pharmacokinetics and Bioavailability; and Labeling Documentation” [60,61].

Liposomes can be engineered to release encapsulated drugs through various mechanisms depending on the intended therapeutic application. In this study, the mild-hyperthermia method, which enhances liposomal permeability by increasing temperature, was employed. This method is not only recognized as an effective liposomal drug release strategy, but is also therapeutically relevant in the context of skeletal muscle disorders, where localized heat applications are routinely used to alleviate muscle pain and support tissue regeneration [62,63,64,65,66,67,68]. Experimental results revealed that the release rates of NMN and Matrigel were lower when encapsulated individually in liposomes compared with the co-release from a therapeutic mix encapsulated within the liposomal core. This suggests a synergistic effect between the two compounds. Thus, a hypothesis supported by the results of this study is that NMN diffusion across the liposomal membrane generates an osmotic gradient that facilitates the co-transport of larger molecules such as Matrigel. Additionally, given the complex structure of Matrigel and as above-mentioned, it is plausible that a part remains adsorbed on the liposomal surface rather than being fully encapsulated. As NMN diffuses, it may induce membrane dynamics to dislodge and release surface-bound Matrigel elements. We acknowledge that further studies would be necessary to validate the proposed mechanism of NMN-mediated enhancement of release kinetics.

Matrigel is a complex mixture of proteins and proteoglycans that naturally tend to form fibrillar networks, while NMN is highly hydrophilic, facilitating diffusion through the lipid bilayer. The results indicate that NMN enhances the diffusion of Matrigel but in a controlled manner, ensuring a more sustained release profile of the therapeutic mix. Although certain limitations exist in using Matrigel for sarcopenia treatment when evaluating its advantages versus disadvantages, the benefits outweigh the challenges. Matrigel is widely utilized in preclinical research and cell culture applications due to its strong capacity to support cell proliferation and tissue-like microenvironments. However, it is important to acknowledge certain limitations associated with its use. These include batch-to-batch variability, a poorly defined molecular composition, and its origin from murine sarcoma tissue, factors that may limit its applicability in clinical research and translational medicine due to concerns about reproducibility and regulatory acceptance [69,70]. As a result, Matrigel remains a biologically relevant material in the effort to combat muscle atrophy associated with sarcopenia.

A study by Kitora Dohi et al. [71] investigated the potential of extracellular matrix-based cell therapy for treating muscle atrophy. The research team isolated the extensor digitorum longus muscle from mice, separated individual muscle fibers, and extracted satellite cells, which were then expanded and differentiated into myoblasts over seven to eight days. The myoblasts were then centrifuged and mixed with Matrigel at varying concentrations of 0.5, 2.5, and 5.0 mg/mL. This mixture was injected into the tibialis anterior muscle following an induced muscle injury under a standardized protocol.

After evaluating the results, the study demonstrated that Matrigel supported muscle tissue integrity following transplantation, leading to increased muscle mass and improved muscle structure. The study provides strong evidence that combining myoblasts with Matrigel enhances muscle regeneration, making it a potential therapeutic approach for sarcopenia. To investigate the protective potential of DMPC-based lipid vesicles against sarcopenia-associated oxidative stress, mitochondrial dysfunction, and altered myotube morphology, H_2_O_2_ was used to mimic in vitro the pathophysiological features of sarcopenic myotubes [72]. The obtained results demonstrated that the exposure of C2C12 myotubes to H_2_O_2_ triggered pronounced oxidative stress, evidenced by a marked reduction in cell viability, elevated levels of ROS and NO, loss of mitochondrial membrane potential, and typical cytoskeletal architecture. These findings confirm the successful establishment of a consistent and reproducible in vitro model of sarcopenia [73,74]. Treatment with DMPC vesicles alone did not alleviate any of the H_2_O_2_-induced damage, as all of the measured biological parameters remained similar to those observed in the sarcopenia-mimicking group. These results indicate that the pristine lipid DMPC vesicles lack intrinsic bioactivity under oxidative conditions and are ineffective as standalone therapeutic tools. The DMPC_Mgel group showed similar poor outcomes in the biological assessments, showing no significant improvement in cell viability, ROS and NO production, or mitochondrial membrane potential compared with the H_2_O_2_-treated group. However, exposed to DMPC_Mgel treatment, the myotubes displayed a modest but noteworthy improvement in cytoskeletal organization, with some cells exhibiting more continuous and aligned F-actin filaments than in the H_2_O_2_ and DMPC_Ctrl-treated groups. This partial structural preservation was most likely due to the Matrigel’s extracellular matrix components, which play a key role in supporting cellular adhesion and mechanical stability [75,76]. While Matrigel did not confer biochemical protection, its presence may have contributed to maintaining cellular architecture under stress conditions. In contrast, the addition of NMN in the lipid formulations significantly enhanced the protective effect against sarcopenia damage. Compared with the H_2_O_2_-treated group, the DMPC_NMN treated myotubes displayed significantly improved cell viability, reduced oxidative and inflammatory stress, and partial restoration of the mitochondrial membrane potential. Moreover, the cytoskeletal architecture was substantially recovered, with elongated and well-aligned F-actin filaments. These results are consistent with NMN’s established role in supporting mitochondrial function and counteracting oxidative stress [77,78,79]. Notably, the combined DMPC_NMN_Mgel formulation yielded the most consistent and pronounced protective effects, as all of the investigated biochemical parameter levels were comparable to the control, together with a nearly complete preservation of the cytoskeletal architecture. These findings support a synergistic interaction between NMN and Matrigel, where NMN provides metabolic and antioxidant support, while Matrigel enhances structural integrity. Therefore, the co-delivery of NMN and Matrigel via lipid vesicles represents a promising strategy to counteract sarcopenia-induced damage in muscle cells.

## 4. Materials and Methods

### 4.1. Materials

The preparation of lipid vesicles utilizes 1,2-dimyristoyl-sn-glycero-3-phosphocholine (DMPC), chloroform (CHCl_3_), phosphate-buffered saline (PBS) tablets, and ultrapure water. The successful formulation of these DMPC-based vesicles involves encapsulating nicotinamide mononucleotide (NMN) and Corning^®^ Matrigel^®^ Growth Factor Reduced (GFR) Basement Membrane Matrix (Mgel). DMPC was sourced from Avanti Polar Lipids (Sigma Aldrich/Merck, Burlington, MA, USA), while the CHCl_3_ and PBS tablets were obtained from Sigma Aldrich/Merck (Burlington, MA, USA). High-purity NMN was procured from a local supplement store, and Mgel was acquired from Corning (Sigma Aldrich/Merck, Burlington, MA, USA).

### 4.2. Methods

#### 4.2.1. Synthesis of DMPC-Based Lipid Vesicles Encapsulate NMN and Matrigel

The initial step involved dissolving DMPC in chloroform, maintaining in each sample an amount of 4 mg of DMPC with a DMPC:CHCl_3_ w:v ratio of 1:4. This mixture was placed in a round-bottom flask connected to a rotary evaporator, operated at a water bath temperature of 42 °C—aligning with the specific melting temperature (Tm) of DMPC—while set to 60 rpm and low pressure (500 mbar). Once the chloroform had evaporated, a dry lipid film of DMPC was formed, which was then hydrated with PBS for 2 h in an ultrasound bath, resulting in a cloudy stock solution. To effectively form lipid vesicles, an ultrasound probe was employed with the following parameters: an operating time of 3 min, alternating between 20 s on and 5 s off, and an amplitude of 25%.

#### 4.2.2. Control Sample (DMPC_Ctrl)

A part of the DMPC stock solution was sonicated under the aforementioned conditions without any additional components. This sample served as the control (DMPC_Ctrl).

#### 4.2.3. NMN-Loaded Vesicles (DMPC_NMN)

The same procedure was followed, but nicotinamide mononucleotide (NMN) was added to the DMPC solution before sonication, achieving a final NMN concentration of 250 µg/mL. This sample was coded as DMPC_NMN.

#### 4.2.4. Matrigel-Loaded Vesicles (DMPC_Mgel)

Due to Matrigel’s temperature sensitivity (transitioning from liquid to gel at room temperature), the process required an ice flake bath as a cooling support. The DMPC stock solution was placed on ice, and Matrigel was added using micropipette tips pre-cooled in a refrigerator to ensure precise handling. The ultrasound probe was then rapidly activated to facilitate Matrigel encapsulation. The final concentration of Matrigel was 250 µg/mL, and this sample was designated as DMPC_Mgel.

#### 4.2.5. Dual-Encapsulated Vesicles (DMPC_NMN_Mgel)

The fourth sample followed the same synthesis process as DMPC_Mgel but included both NMN and Matrigel at equal concentrations (250 µg/mL each). This sample was referred to as DMPC_NMN_Mgel.

As lipid vesicle formation progressed, the turbidity of the solutions gradually decreased. After preparation, all four DMPC-based samples were stored in a refrigerator and subsequently analyzed for their physico-chemical and biological properties.

### 4.3. Analysis Methods

#### 4.3.1. Dynamic Light Scattering (DLS)

Lipid vesicle samples were analyzed using DLS to determine their hydrodynamic diameter, zeta potential, and polydispersity index. These parameters are critical for evaluating the stability and size distribution of lipid vesicles, particularly in the context of medical drug delivery applications, where the solution stability and size consistency are essential. The measurements were conducted using a DelsaMax Pro (Beckman Coulter, Brea, CA, USA). Each sample was injected into the instrument’s measurement cell, with PBS as the solvent. To ensure accuracy and reproducibility, all measurements were performed in triplicate. Data are presented as the mean ± standard deviation.

#### 4.3.2. Scanning Electron Microscopy (SEM)

The morphological and dimensional properties of the lipid vesicles were examined using SEM analysis. A micropipette was utilized to apply 10 µL of each sample onto a carbon strip, which was then placed in a dust-free environment to allow for gradual, natural drying at room temperature. This method was employed to avoid any alterations to the characteristics of the lipid vesicles. The analysis was conducted using a Versa 3D focused ion beam-scanning electron microscope (FIB-SEM) from Thermo Fisher Scientific (Waltham, MA, USA). High-resolution micrographs were obtained by detecting secondary electron (SE) signals at 10 keV using the electron detector within the microscope.

#### 4.3.3. Transmission Electron Microscopy (TEM)

The samples were further analyzed using TEM microscopy to gain additional insights into the morphology and dimensions of the lipid vesicles as well as to assess the integrity of their membranes. Ten microliters of each sample was placed onto a 400 mesh lacey carbon-coated copper grid. The grids were allowed to dry at room temperature in a controlled manner to preserve the vesicle characteristics. The analysis was conducted using a TECNAI F30 G2 S-TWIN microscope (FEI, Hillsboro, Oregon, USA), which provided high-resolution micrographs essential for evaluating the morphological and dimensional properties of lipid vesicles.

#### 4.3.4. Encapsulation Efficiency (EE%)

The purpose of this evaluation was to separate lipid vesicles from the unencapsulated drug to assess the encapsulation efficiency (EE%). For this analysis, UV–Vis spectroscopy was utilized, employing a Thermo Fisher Scientific Evolution 300 double-beam spectrophotometer (Waltham, MA, USA) for all spectrophotometric measurements. Data processing was conducted with the VisionPro software (version 4.5.0). To separate the encapsulated drug from the unencapsulated drug, equal volumes of the samples were centrifuged at 8500 rpm for 2 h. The supernatant, which contained the unencapsulated drug, was then collected for analysis. The concentration of the unencapsulated drugs was quantified using a five-point calibration curve established across a range of concentrations from 25 to 200 µg/mL for both NMN and Matrigel. For the evaluation of Matrigel, bovine serum albumin (BSA) was used as a reference due to its similar protein composition, which absorbs at the same wavelength. Spectral measurements were taken in the wavelength range of 190–350 nm, with NMN displaying a characteristic maximum absorption at 260 nm and BSA at 280 nm. The resulting calibration curves exhibited excellent linearity, with correlation coefficients (R^2^ values) of 0.9999 for NMN and 0.9996 for BSA. Using these spectral measurements, the encapsulation efficiency (EE%) of the lipid vesicles was calculated using the following formula:

$$EE\%=\left(\frac{C_{total}-C_{free}}{C_{total}}\right)\times 100$$

$C_{total}$ represents the initial concentration of the drug added, while $C_{free}$ denotes the concentration of the unencapsulated drug.

#### 4.3.5. Drug Release %

After separating the lipid vesicles from the unencapsulated drugs, the pellet obtained from each of the three samples following centrifugation was redispersed in PBS and incubated in a water bath at 40 °C for 8 h. Samples were collected at predetermined time intervals of 5, 30, 60, 180, 300, and 480 min. To maintain consistency throughout the experiment, an equivalent volume of PBS was added to the samples after each collection to ensure uniform conditions. For the evaluation of drug release, UV-Vis spectroscopy was employed, utilizing the same equipment and calibration curves established previously for encapsulation efficiency assessment. The spectral measurements obtained allowed for the construction of the drug release profiles for the three formulations tested, calculated using the following formula:

$$Drug\;release\;\%=\left(\frac{C_{release}}{C_{encapsulated}}\right)\times 100$$

where $C_{release}$ denotes the drug concentration released over time, and $C_{encapsulated}$ represents the concentration of the encapsulated drug.

### 4.4. In Vitro Biological Investigations

#### 4.4.1. Cell Culture Model

The murine myoblast cell line C2C12 (ATCC CRL-1772) was employed as an in vitro model system for all of the experimental procedures. Cells were cultured in Dulbecco’s modified Eagle’s medium (DMEM; Sigma-Aldrich), enriched with 10% fetal bovine serum (FBS; Gibco, Thermo Fisher Scientific, Waltham, MA, USA) and 1% antibiotic-antimycotic solution (ABAM; Sigma-Aldrich). Cultures were maintained in a humidified incubator at 37 °C with 5% CO_2_ atmosphere.

Myogenic differentiation was initiated to generate mature myotubes for further experiments. For this purpose, C2C12 cells were plated and allowed to reach approximately 80% confluency. At this point, cell differentiation was induced by replacing the standard growth medium with high-glucose DMEM supplemented with 2% horse serum (HS; Sigma-Aldrich). Cells were then cultured under the same incubation conditions for four days to achieve mature myotubes that were subsequently allocated into the following experimental conditions:

A. Control group—differentiation medium was refreshed without additional treatment;
B. Sarcopenia model group—myotubes exposed to 100 μM H_2_O_2_ to induce a sarcopenia-like phenotype;
C. DMPC group—myotubes treated with 100 μM H_2_O_2_ + DMPC vesicles;
D. DMPC NMN group—myotubes treated with 100 μM H_2_O_2_ + DMPC vesicles loaded with NMN;
E. DMPC Matrigel group—myotubes treated with 100 μM H_2_O_2_ + DMPC vesicles loaded with Matrigel;
F. DMPC NMN + Matrigel group—myotubes treated with 100 μM H_2_O_2_ + DMPC vesicles loaded with NMN and Matrigel.

Cells were cultured for 48 h under these conditions, after which multiple biochemical and cellular investigations were performed to assess the treatment cytotoxicity, mitochondrial function, oxidative stress, and cellular structure.

#### 4.4.2. Cell Viability Evaluation

To determine the effects of the tested treatments on the viability of differentiated myotubes, the MTT assay was employed. After the cell culture medium was discarded, cells were incubated with a 1 mg/mL MTT solution (Sigma Aldrich) prepared in serum-free DMEM for 4 h at 37 °C. Following incubation, the insoluble formazan crystals formed by mitochondrial reduction were dissolved using isopropanol. Absorbance was subsequently measured at 550 nm with the FlexStation 3 microplate reader (Molecular Devices, San Jose, CA, USA). Cell viability was expressed as a percentage of the control group using the formula: Cell viability (%) = (Absorbance of treated cells/Absorbance of control cells) × 100.

#### 4.4.3. Detection of Intracellular Reactive Oxygen Species (ROS)

ROS production was evaluated using the fluorescent dye 2′,7′-dichlorofluorescein diacetate (DCFH-DA, Sigma-Aldrich). Following 48 h of treatment, cells were rinsed with PBS and incubated in 10 µM DCFH-DA prepared in serum-free DMEM for 30 min at 37 °C, protected from light to minimize photooxidation. Unbound dye was removed by PBS washing, and fluorescence intensity was measured immediately at an excitation/emission wavelength of 485/530 nm using a FlexStation 3 reader. Data were normalized to the control levels and reported as a percentage: ROS (%) = (Fluorescence of treated cells/Fluorescence of control cells) × 100.

#### 4.4.4. Nitric Oxide (NO) Quantification

Nitric oxide levels in the culture medium were determined using the Griess Reagent System (Promega, Madison, WI, USA), following the supplier’s instructions. Supernatants (50 μL) were transferred to a 96-well plate and reacted with an equal volume of sulfanilamide solution for 10 min at room temperature in the dark. This was followed by the addition of 50 μL NED solution and a second 10-min incubation step. The resulting colorimetric signal was measured at 540 nm using the FlexStation 3. A standard curve generated with sodium nitrite (0–100 μM) was used to calculate the nitrite concentrations, which were expressed in μM per sample.

#### 4.4.5. Mitochondrial Membrane Potential (MMP) Evaluation

Mitochondrial membrane potential was assessed using JC-10 dye (MAK159 Kit, Sigma-Aldrich), a dual-emission fluorescent probe that differentiates between polarized and depolarized mitochondria. After 48 h of treatment, 50 µL of the JC-10 working solution (prepared by diluting the stock in Assay Buffer A) was added to each well and incubated for 45 min at 37 °C in a 5% CO_2_ atmosphere, shielded from light. After incubation, 50 µL of Assay Buffer B was added. Fluorescence was measured at excitation/emission 540/590 nm for the JC-10 aggregates (red, polarized) and 490/525 nm for monomers (green, depolarized). The MMP was represented as the red-to-green fluorescence ratio: MMP = Red (590 nm)/Green (525 nm).

#### 4.4.6. Analysis of Cytoskeletal Integrity

F-actin organization was visualized using TRITC-conjugated phalloidin staining (Sigma-Aldrich). Cells were fixed in 4% paraformaldehyde for 15 min and permeabilized with a blocking buffer containing 2% BSA and 0.1% Triton X-100 for 10 min at room temperature. Staining was performed with TRITC-phalloidin (1:100 dilution) for 1 h at 37 °C, protected from light. Nuclear counterstaining was achieved using DAPI (Sigma-Aldrich) for 20 min. Fluorescence images were acquired using an Olympus IX73 microscope with CellSense F software (version 8.0.2), and the cytoskeletal morphology was further analyzed using ImageJ (version 1.53, NIH).

#### 4.4.7. Statistical Analysis

All experiments were independently conducted in triplicate. Data are presented as the mean ± standard deviation (SD). Statistical analyses were performed using GraphPad Prism version 9, with significance set at *p* < 0.05. Biological replicates were used unless otherwise stated. For comparisons between the two groups, unpaired two-tailed Student’s *t*-tests were used. For comparisons among more than two groups, one-way ANOVA followed by Tukey’s post hoc test was applied.

## 5. Conclusions

This study proposed a novel approach to managing symptoms associated with sarcopenia and preventing its early onset by developing and testing liposome-based vesicles designed to encapsulate a therapeutic mix selected based on deficiencies that contribute to the progression and severity of the condition. The findings revealed that the physicochemical properties of these liposomal formulations were influenced both by the synthesis process and the type of therapeutic substance encapsulated. A notable synergistic effect was observed between the chemical compound NMN and the biological component Matrigel, resulting in the DMPC_NMN_Mgel formulation exhibiting the optimal dimensional characteristics, well-defined spherical morphologies, and a controlled, time-dependent drug release profile, making it highly suitable for the intended application. The role of NMN in anti-sarcopenia therapies has been extensively studied, with numerous reports highlighting its promising effects in muscle preservation and regeneration. In contrast, the therapeutic potential of Matrigel in this context has only been explored in a limited number of studies including the present one. However, the results consistently indicated that Matrigel exerts significant effects on muscle tissue regeneration, suggesting that it may play a crucial role in future sarcopenia management strategies. This research further reinforces the importance of exploring liposomal drug delivery systems as a versatile and efficient tool for addressing muscle degeneration associated with aging and muscle-wasting conditions. Results suggest that liposomal delivery enhances the bioavailability and retention time, providing a sustained therapeutic effect. Given Matrigel’s role in muscle repair and NMN’s ability to boost mitochondrial function, their co-encapsulation in DMPC liposomes offers a possible nanomedicine strategy for sarcopenia treatment, based on the tests conducted thus far, so further validation through in vivo evaluation remains necessary.

## Figures and Tables

**Table 1:** Data of hydrodynamic diameter (nm) as a DLS result of DMPC vesicles dispersed in PBS.

	DMPC_Ctrl	DMPC_NMN	DMPC_Mgel	DMPC_NMN_Mgel
Hydrodynamic diameter (nm)	163.6	196.4	228.8	267.4

**Table 2:** Data of zeta potential (mV) as a DLS result of DMPC vesicles dispersed in PBS.

	DMPC_Ctrl	DMPC_NMN	DMPC_Mgel	DMPC_NMN_Mgel
Zeta potential (mV)	−11.27	−8.42	−5.58	−5.55

**Table 3:** PDI data as a DLS result of DMPC vesicles dispersed in PBS.

	DMPC_Ctrl	DMPC_NMN	DMPC_Mgel	DMPC_NMN_Mgel	PDI Range
PDI	0.081	0.081	0.081	0.081	*<0.1 (Monodisperse)*

## Data Availability

At authors, by request.
